# A Potential Role for Epigenetic Processes in the Acclimation Response to Elevated *p*CO_2_ in the Model Diatom *Phaeodactylum tricornutum*

**DOI:** 10.3389/fmicb.2018.03342

**Published:** 2019-01-14

**Authors:** Ruiping Huang, Jiancheng Ding, Kunshan Gao, Maria Helena Cruz de Carvalho, Leila Tirichine, Chris Bowler, Xin Lin

**Affiliations:** ^1^State Key Laboratory of Marine Environmental Science,College of Ocean and Earth Sciences, Xiamen University, Xiamen, China; ^2^School of Pharmaceutical Sciences, Xiamen University, Xiamen, China; ^3^Ecology and Evolutionary Biology Section, Institut de Biologie de l’École Normale Supérieure (IBENS), Département de Biologie, Ecole Normale Supérieure, CNRS UMR8197, Inserm U1024, PSL Research University, Paris, France; ^4^Faculté des Sciences et Technologie, Université Paris Est-Créteil, Créteil, France; ^5^Faculté des Sciences et Techniques, Université de Nantes, CNRS UMR6286, UFIP, Nantes, France

**Keywords:** ocean acidification, climate change, diatom, transposable element, histone, long non-coding RNA

## Abstract

Understanding of the molecular responses underpinning diatom responses to ocean acidification is fundamental for predicting how important primary producers will be shaped by the continuous rise in atmospheric CO_2_. In this study, we have analyzed global transcriptomic changes of the model diatom *Phaeodactylum tricornutum* following growth for 15 generations in elevated *p*CO_2_ by strand-specific RNA sequencing (ssRNA-seq). Our results indicate that no significant effects of elevated *p*CO_2_ and associated carbonate chemistry changes on the physiological performance of the cells were observed after 15 generations whereas the expression of genes encoding histones and other genes involved in chromatin structure were significantly down-regulated, while the expression of transposable elements (TEs) and genes encoding histone acetylation enzymes were significantly up-regulated. Furthermore, we identified a series of long non-protein coding RNAs (lncRNAs) specifically responsive to elevated *p*CO_2_, suggesting putative regulatory roles for these largely uncharacterized genome components. Taken together, our integrative analyses reveal that epigenetic elements such as TEs, histone modifications and lncRNAs may have important roles in the acclimation of diatoms to elevated *p*CO_2_ over short time scales and thus may influence longer term adaptive processes in response to progressive ocean acidification.

## Introduction

Ocean acidification is caused by the absorption of excessive anthropogenic CO_2_ emissions by the ocean. It is predicted that the further rise of atmospheric CO_2_ concentrations under a “business-as-usual” CO_2_ emission scenario will lead to a further drop of ocean surface pH by 0.4 units by 2100 (Gattuso et al., 2015). Diatoms, responsible for about 20% of global primary production, profoundly influence marine food webs and biogeochemical cycles (Tréguer et al., 2018). Although several studies reporting the effects of ocean acidification on diatoms at the physiological and ecological levels have been reported (Gao and Campbell, 2014), there are only limited studies deciphering the underlying molecular mechanisms of diatoms in response to ocean acidification.

Most studies that have examined the genetic responses of diatoms to ocean acidification have focused on carbon fixation and assimilation genes. It has been reported that the large subunit of ribulose-1, 5-bisphosphate carboxylase/oxygenase (Rubisco), involved in the first committed step of carbon fixation, was down-regulated at the transcriptional and protein levels under elevated *p*CO_2_ conditions (Losh et al., 2013; Li et al., 2017). Genes encoding components of carbon concentrating mechanisms (CCMs) including carbonic anhydrases and bicarbonate transporters were also reported to be down-regulated under elevated CO_2_ conditions (Hopkinson et al., 2011; Li et al., 2014; Hennon et al., 2015). Furthermore, using RNA-seq in the context of a nitrate-limited chemostat experiment, it was additionally reported that elevated CO_2_ first caused changes in transcriptional regulation and chromosome folding and subsequently metabolic rearrangements in *Thalassiosira pseudonana*. This study further revealed that genes in one CO_2_-responsive cluster share a putative cAMP responsive *cis-*regulatory sequence for a cAMP second messenger system (Hennon et al., 2015). These results imply that in addition to genes encoding central components of carbon fixation and assimilation, the response to elevated CO_2_ may involve a broader reprogramming of transcription, genome structure, and cell signaling.

Long non-protein coding RNAs (lncRNAs) are transcripts longer than 200 nucleotides which are similar to mRNAs but have low to no apparent protein coding potential. Long considered as transcriptional noise, numerous evidence from animals and plants gathered over recent years indicate that lncRNAs have multiple and important functions, such as histone modification regulation, transcription machinery regulation and post-translational regulation (Lee, 2012; Hon et al., 2017). Furthermore, lncRNAs are often associated with stress responses having fundamental regulatory functions in cellular adaptation and homeostasis (Amaral et al., 2013). LncRNAs may also have important roles in diatoms in response to different environmental stimuli, however, to our knowledge only one study has so far investigated changes in lncRNA levels in response to phosphate fluctuations (Cruz de Carvalho et al., 2016).

Histones play crucial roles in chromosome structure and genome stability. A total of 14 histone genes (H2A, H2B, H3, H4, and H1) are dispersed on 5 of the 34 chromosome scaffolds of the *P. tricornutum* genome (Bowler et al., 2008). Transposable elements (TEs) are mobile DNA sequences able to move in the genome by generation of new copies. TEs are ancient and active genomic components which have been found in most eukaryotic and prokaryotic genomes (Feschotte and Pritham, 2007). In *P. tricornutum* and *T. pseudonana*, 6.4 and 1.9% of the genome are contributed by TEs, respectively (Armbrust et al., 2004; Bowler et al., 2008; Rastogi et al., 2018). TEs are classified into retrotransposons and DNA transposons according to the presence or absence of an RNA transposition. Retrotransposons use a “copy and paste” strategy to proliferate in the genome whereas the majority of DNA transposons use a “cut and paste” strategy to move in the genome. A total of 90% and 58% of TEs are long terminal repeat retrotransposons (LTR-RTs) in the *P. tricornutum* and *T. pseudonana* genomes, respectively. Among LTR-RTs, *gag pol* domains are the most abundant TEs in *P. tricornutum* (Maumus et al., 2009; Rastogi et al., 2018). Retrotransposons are considered as the major contributors to the expansion of genomes especially in higher eukaryotes and enhance their hosts’ fitness by advantageous genome re-arrangements in a changing environment (Schaack et al., 2010; Casacuberta and González, 2013).

In this study, we have combined physiological performance measurements, with strand-specific RNA sequencing (ssRNA-Seq) to assess global transcriptomic changes in *P. tricornutum* in response to elevated *p*CO_2_ with respect to ambient *p*CO_2_ concentrations following growth for 15 generations. Our data provide new insights into how a diatom copes with elevated *p*CO_2_ using some potential epigenetic mechanisms and suggest an involvement of TEs and possibly lncRNAs in the acclimation response to elevated *p*CO_2_.

## Materials and Methods

### *P. tricornutum* Culture Conditions and Physiological Measurements

Axenic cultures of *Phaeodactylum tricornutum* (Bohlin) strain CCMP 632 were obtained from the Culture Center of Marine Phytoplankton (East Boothbay, ME, United States). *P. tricornutum* cells were cultured in 2L polycarbonate bottles (Nalgene, United States) containing 1.7–1.8 L artificial seawater enriched with Aquil medium at 20°C under cool white fluorescent lights at 150 mmol m^-2^ s^-1^ (12 h:12 h light: dark). Different *p*CO_2_ in the medium was achieved by bubbling different concentrations of CO_2_. The gas streams were pre-filtered through 0.2-μm HEPA filters at a flow rate of 0.6 L min^-1^. The low CO_2_ medium was bubbled with ambient air of about 400 ppmv (low CO_2_, LC) and the high CO_2_ medium was bubbled with pre-mixed air-CO_2_ mixtures (1000 ppmv; high CO_2_, HC) from a plant growth CO_2_ chamber (HP1000G-D, Ruihua), controlling the CO_2_ concentration with a less than 3% variation. Triplicates of LC and HC cultures were all started with the same initial cell densities of 2 × 10^4^ cells mL^-1^. The culture was refreshed with *p*CO_2_ and the pH adjusted medium every 48 h to maintain the desired pH and *p*CO_2_ values within a variation of less than 0.5%. The carbonate system in the HC cultures differed significantly from the LC culture (Supplementary Table S1). The growth rate was calculated as μ = (ln *C*_1_ - ln *C*_0_)/(*t*_1_ -*t*_0_), where *C*_0_ and *C*_1_ represent the cell density at *t*_0_ (initial or just after the dilution) and *t*_1_ (before the dilution), respectively. Cell densities were measured by particle count and size analyzer (Z2 Coulter, Beckman). Pigment concentrations were measured as described in (Porra et al., 1989) using spectrophotometry (Beckman DU-800, United States). The maximum quantum yield (*F*_v_/*F*_m_), effective quantum yield (Yield) were measured by a Xenon-Pulse Amplitude Modulated Fluorometer (XE-PAM, Walz, Germany) at the middle of the light period. Particulate organic nitrogen (PON) and carbon (POC) were measured according to (Liu et al., 2017).

### RNA Extraction, Library Preparation and ssRNA-seq

*Phaeodactylum tricornutum* cells from three biological replicates were harvested in the middle of light period by vacuum filtration on 2 mm polycarbonate filters (Millipore, United States) following 15 generations (day 10), flash frozen in liquid N_2_ and maintained at -80°C until use. Total RNA was extracted from frozen cell pellets using TRIzol^®^ Reagent (Invitrogen, Carlsbad, CA, United States) according to the manufacturer’s instructions. RNA integrity was assessed using the RNA Nano 6000 Assay Kit of the 2100 Bioanalyzer (Agilent Technologies, CA, United States) and by RT-qPCR. A total amount of 3 μg RNA per sample was used for construction of RNA sample libraries. Ribosomal RNA was removed by Epicentre Ribo-Zero^TM^ Gold Kits (Epicentre, United States). The sequencing libraries were generated using NEBNext Ultra Directional RNA Library Prep Kit (NEB, Ipswich, United States) with varied index labeling following the manufacturer’s guidelines. After cluster generation by HiSeq PE Cluster Kit v4-cBot-HS (Illumina), the libraries were sequenced on an Illumina Hiseq 4000 platform and 150 bp paired-end reads were generated. Library preparation and ssRNA-seq were performed offsite at Annoroad Gene Technology Corporation (Beijing, PR China).

### ssRNA-seq Data Assembly and Analysis

Raw data from ssRNA-seq were cleaned up by removing adapter-contaminated reads (more than 5 adapter-contaminated bases), poly-N containing (more than 5%) and low quality reads (quality value less than 19, accounting for more than 15% of total bases). All the downstream analyses were based on filtered clean data with high quality. The average clean data was 7.1 Gb for ssRNA-Seq. The reference genome of *P. tricornutum* was downloaded^1^. Bowtie2 v2.2.3 was used for building the genome index (Langmead et al., 2009), and clean data was mapped to the reference genome using TopHat v2.0.12 (Trapnell et al., 2009). Read counts for each gene in each sample were counted by HTSeq v0.6.0, and RPKM (Reads Per Kilobase Million Mapped Reads) was then calculated to estimate the expression level of genes in each sample (Wagner et al., 2012).

DESeq (v1.16) was used for differential gene expression analysis. The *p*-value was assigned to each gene and adjusted by the Benjamini and Hochberg’s approach for controlling the false discovery rate. Genes with fold change ratio (HC/LC) ≥2 (*p*_adj_ ≤ 0.05) and fold change ratio ≤0.5 (*p*_adj_ ≤ 0.05) were defined as “up-regulated genes” and “down-regulated genes,” respectively. Genes with fold change ratio (HC/LC) between 1.5 and 2 (*p*_adj_), fold change between 0.5 and 0.67 (*p*_adj_) were defined as “relatively up-regulated genes” and “relatively down-regulated genes,” respectively.

### GO and KEGG Analysis

Gene Ontology analysis^2^ was conducted to construct meaningful annotation of genes and gene products in different databases and species. GO enrichment analysis was used to assess the significance of GO term enrichment among differentially expressed genes in the domains of biological processes, cellular components and molecular functions. The GO enrichment of differentially expressed genes was implemented by the hypergeometric test, in which *p*-value was calculated and adjusted as *q*-value. GO terms with *q* < 0.05 were considered to be significantly enriched. We further performed KEGG^3^ pathway analysis to explore the roles of the differentially expressed genes in different metabolic pathways and molecular interactions. The genes related to chromatin, protein DNA, chromosomal part, chromosome, histone modification, glycolysis, tricarboxylic acid cycle (TCA cycle), and photosynthesis (light part), carbon assimilation and N uptake and assimilation were classified manually based on the KEGG and GO gene classification (Supplementary Table S2).

### Identification of Novel lncRNAs

LncRNA candidates with length of ≥200 nucleotides, a predicted open reading frame (ORF) of ≤100 amino acids, number of exon ≥ 1 and reads ≥ 3 were selected (Liu et al., 2012; Cruz de Carvalho et al., 2016). Transdecoder was used to remove known mRNA transcripts and candidates with protein coding potential. The novel lncRNAs were classified based on the location of the lncRNA gene region in the reference genome. In order to increase the depth of the reads for the increased detection of non-coding RNA transcripts, the non-coding RNA transcript reads derived from the three replicates were pooled since the Pearson correlation coefficients (PCCs) between the RPKM of three replicates of LC and HCs were high (>0.97). A 1.5-fold/0.67-fold (up-regulated/down regulated) and variance in RPKM and a *p*-value (Fisher’s exact test) of <0.05 were used as cutoffs to define differentially expressed genes, respectively.

### Interaction Network Construction

The *P. tricornutum* protein–protein interaction network data was downloaded^4^. The differentially expressed mRNA interaction networks, specifically transcription factor (TF), histone modification genes and mRNAs interaction networks were constructed in Cytoscape 3.4.0 based on the *P. tricornutum* protein–protein interaction network data database.

### Quantitative Real-Time Reverse Transcription PCR

Reverse transcription quantitative PCR (RT-qPCR) was used to verify the transcriptomic data. The primers used for qPCR are listed in Supplementary Table S3. Polyadenylated RNA was converted to cDNA by PrimeScript^TM^ II Reverse Transcriptase (Takara). The RT product was then used as the template for qPCR. All qPCR reactions were performed on a FTC2000 (Canada) using SYBR Green I mix (Takara) in 96-well plates according to the manufacturer’s recommendations. TBP (TATA box binding protein) and RPS (ribosomal protein small subunit 30S), two housekeeping genes (Siaut et al., 2007), were used as references to calibrate the expression. Three technical and three biological replicates were conducted.

## Results and Discussion

### Physiological Response of *P. tricornutum* to Elevated *p*CO_2_

No significant differences were detected between cells grown under the HC and LC conditions with respect to pigment contents, *F*_v_/*F*_m_, photosynthetic yield, POC and PON content after growth for 15 generations (Table 1). However, a slight increase in specific growth rate (2.6%) was observed in the HC condition, which is consistent with the previous study (Wu et al., 2010). And the pigment contents, photosynthetic efficiency and growth rate were not significantly different between LC and HC cultures grown for 2 days (∼2.7 generations). The lack of physiological differences between LC and HC suggests an effective and stable acclimated state to the new CO_2_ saturated environment, revealing a very dynamic plasticity to environmental change in diatoms. The underlying transcriptomic changes may help to maintain the physiological performance in response to elevated CO_2_.

**Table 1:** The physiological parameters of *Phaeodactylum tricornutum* after growing for 15 generations.

	μ	Pigment contents (pg cell^-1^)			Photosynthetic efficiency		Partical organic matter contents (pg cell^-1^)	
				
		Chl a	Chl c	Carotenoid	*F*_v_/*F*_m_	Yield	POC	PON
LC	0.98 ± 0.01^a^	0.23 ± 0.01^a^	0.39 ± 0.01^a^	0.18 ± 0.01^a^	0.64 ± 0.01^a^	0.55 ± 0.01^a^	13.41 ± 0.48^a^	2.43 ± 0.14^a^
HC	1.01 ± 0.01^b^	0.23 ± 0.01^a^	0.40 ± 0.01^a^	0.18 ± 0.00^a^	0.62 ± 0.01^a^	0.56 ± 0.01^a^	12.33 ± 0.66^a^	2.27 ± 0.12^a^


*The values are means ± SD, and the different letters represents significant differences between LC and HC grown cells*.

### The Global Transcriptomic Response to Elevated *p*CO_2_ After Growing for 15 Generations

ssRNA-seq data generated from the HC and LC physiological states were assembled and mapped. The average mapping rate for ssRNA-seq data was about 76.8%. The average mapping rate on the intergenic regions was 24.8% (Supplementary Table S4). The expression profile of each gene is listed in Supplementary Table S5. The GO enrichment analysis of differentially expressed genes in the domains of biological processes, cellular components and molecular functions is shown in Supplementary Figure S1. Globally, 954 protein coding genes were differentially expressed in response to HC with 179 up-regulated genes, 287 relatively up-regulated genes, 175 down-regulated genes and 313 relatively down-regulated genes compared with the LC condition over 15 generations. In general, the numbers of differentially regulated genes under the HC condition were much lower than induced by other stimuli such as nitrogen starvation and phosphate depletion (Ge et al., 2014; Cruz de Carvalho et al., 2016). This may suggest that elevated *p*CO_2_ is not a strong environmental stimulus compared to nitrogen starvation and phosphate depletion for *P. tricornutum*. A good correlation was found between transcriptomic data and RT-qPCR, thus validating the robustness of the sequencing data (Supplementary Table S6).

### The Impact of Elevated *p*CO_2_ on Metabolic Genes

**FIGURE 1:**
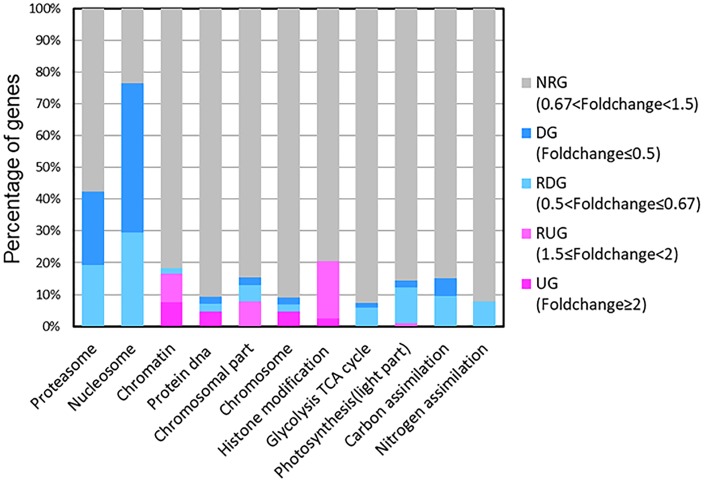
Transcripts abundance (HC/LC) of 11 gene families after *Phaeodactylum tricornutum* grown for 15 generations. Changes are denoted as the percentage of genes up-regulated with fold change ≥ 2 (dark pink, UGs), relatively up-regulated with 1.5 ≤ fold change < 2 (light pink, RUGs), down regulated with fold change ≤ 0.5(dark blue, DGs), relatively down-regulated with 0.5 < fold change ≤ 0.67 (light blue, RDGs) and non-significantly regulated (gray, NRGs) with 0.67 < fold change < 1.5 within each family. The function description of genes in different gene families and their HC/LC fold change can be found in Supplementary Table S4.

Our results showed that some genes involved in CCMs and energy-production/consumption were down-regulated after growing in the HC condition for 15 generations (Figures 1, 5). As expected, some genes involved in CCMs were down-regulated since high CO_2_ is expected to reduce the need for CCMs. Two genes encoding pyrenoid-localized carbonic anhydrases (Pt CA1: Phatr3_J51305, Pt CA2: Phatr3_J45443) (Tachibana et al., 2011) were down-regulated in the HC condition while other genes encoding carbonic anhydrases were not significantly affected by changing CO_2_ concentrations. As reported in (Tachibana et al., 2011; Tanaka et al., 2016), Pt CA1 and Pt CA2 was down-regulated under 5% CO_2_ (50,000 ppmv). Therefore, Pt CA1 and Pt CA2 may be the most responsive carbonic anhydrase gene in face of elevated CO_2_ and essential in converting from HCO_3_^-^ to CO_2_ for carbon fixation by Rubisco. Other CCMs components putative HCO_3_^-^ transporter (SLC4-5, Phatr3_ J54405), putative CO_2_ hydration protein (ChpXY, Phatr3_J38227) and malate dehydrogenase (MDH, Phatr3_J42398) were also down-regulated in HC. Furthermore, some genes encoding proteins with roles in energy-producing metabolic pathways such as glycolysis, oxidative phosphorylation and TCA cycle were preferentially down-regulated. This suggested a general reduction in metabolism with an energy saving status in the HC acclimated state. Genes encoding V-type ATPase subunit F (Phatr3_J31133) and mitochondrial F-type ATPase (Phatr3_J39529), which are directly related to energy consumption and production, respectively, showed down-regulation in HC. The down regulation of genes involved in CCMs and energy turnover rate suggest that a general reduction in metabolism may have contributed to the slightly increased growth rate (2.6%) in HC conditions, which is also consistent with previous experimental and modeling results (Wu et al., 2010; Hopkinson et al., 2011).

The photochemical performance was not significantly influenced after 15 generations under elevated *p*CO_2_; however, some light capture genes such as Fucoxanthin-chlorophyll a-c binding proteins and Fucoxanthin-chlorophyll a/c proteins were down-regulated in the acclimated HC condition (Figure 5). A gene encoding a putative Cyt_b6f_ iron-sulfur subunit (Phatr3_J13358), which is essential for electron transport in photosynthesis, was also down-regulated (Figure 5). The down-regulation of genes encoding light capture and electron transport proteins may result from decrease in energy demands of CCMs and maintaining H^+^ gradient across thylakoid membrane. Although photorespiration was not measured in our study, the putative 2-phosphoglycolate phosphatase gene (Phatr3_Jdraft1186), a key enzyme in the photorespiration pathway, was down-regulated indicating possible decrease in photorespiration rates under the HC condition (Figure 5), which is in line with the previous results in *T. pseudonana* (Hennon et al., 2015).

A significant proportion of ribosome genes were down-regulated in HC reflecting either a decreased capacity for protein synthesis or a low turnover of ribosomal proteins while a significant proportion of genes encoding proteasome core complex were significantly down-regulated by elevated *p*CO_2_ suggesting that the protein degradation system was relatively inactive (Figures 1, 5). All these results indicate a low turnover rate of protein synthesis and degradation, which is somewhat in accordance with the lower energy cost implied by the down-regulation of energy producing genes in HC described above. The down-regulation of ribosome genes in the HC condition was in contrast with the previous study on *T. pseudonana* acclimated to high CO_2_ for 10 days (Hennon et al., 2015), probably because the previous study involved low nitrate concentration.

### The Impact of Elevated *p*CO_2_ on Genes Involved in pH Homeostasis Maintenance

Beside genes directly involved in metabolic pathways being affected by elevated *p*CO_2_, some genes related to cellular pH homeostasis maintenance were also affected by elevated *p*CO_2_. It is predicted that the bulk seawater H^+^ concentration (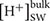) will increase by 100–150% by 2100 in response to ocean acidification (Caldeira and Wickett, 2003). It was reported that the increased pH in seawater results in greater diel variation of H^+^ concentration proximate seawater (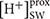) of marine organisms by boundary-layer processes (Caldeira and Wickett, 2003). The changes in 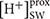 affect the operation of cellular processes involved in pH homeostasis including H^+^ production/consumption and active/passive H^+^ transport (Smith and Raven, 1979; Taylor et al., 2011). It has been reported that the down-regulation of CCMs changes intracellular H^+^ concentration by decreasing HCO_3_^-^ transport into cytoplasmic matrix and conversion from HCO_3_^-^ to CO_2_ (Smith and Raven, 1979). To maintain the neutral and acid pH in cytoplasmic matrix and organelles, the proton pump plays critical roles in H^+^ transport (Smith and Raven, 1979). In our study, Phatr3_J31133 encoding the subunit F of vacuolar V-type proton pump where H^+^ is transported from cytoplasmic matrix into vacuoles coupled with ATP hydrolysis was down-regulated in HC; however, Phatr3_J43207 coding inorganic H^+^ pyrophosphatase was up-regulated stimulating H^+^ transport from cytoplasmic matrix into vacuoles coupled with pyrophosphate hydrolysis (Figure 5). Similarly, Phatr3_J39529 encoding epsilon chain of mitochondrial F-type proton pump in which H^+^ is transported from mitochondria into cytoplasmic matrix coupled with ATP synthesis was down-regulated; however, Phatr3_J50171 encoding protein related to mitochondria K^+^-H^+^ exchange was up-regulated (Figure 5). These results suggest that the decrease in H^+^ transport coupled with ATP metabolism may be offset by other H^+^ transport pathways to maintain the cellular pH homeostasis under high *p*CO_2_ conditions in *P. tricornutum*. More investigations, such as pH measurements in different organelles and the cytoplasm under high *p*CO_2_ conditions compared to ambient conditions, are needed for better understanding of pH homeostasis mechanisms operating under conditions reflecting ocean acidification in diatoms.

### Potential Changes in Epigenetic Regulation in Response to Elevated *p*CO_2_ Over 15 Generations

After growing in HC and LC conditions for 15 generations, the most striking molecular response was the down-regulation of genes encoding histones and the up-regulation of many TEs. TE and histone gene variations and their changes in expression may have fundamental effects on the adaptive responses to elevated *p*CO_2_, as a result stabilizing the physiological performance. 12 out of 14 histone genes (H3, H4, H2B, H3B, and H1) were significantly down-regulated. For example, Phatr3_J54360 encoding H2B 1b isoform showed down-regulation in both RT-qPCR (Supplementary Table S6) and ssRNA-seq data sets in HC. Only Phatr3_J28445 encoding H2A 1 (H2A.Z) and Phatr3_J21239 encoding H3.2 were not significantly affected by elevated *p*CO_2_. Phatr3_EG01358 encoding histone H2A isoform 3a, with fold change 0.098 (HC/LC), was the most significantly down-regulated histone gene. On the other hand, genes encoding core domains of TEs, in particular the *gag pol* genes, were highly expressed in HC compared to LC condition. The LTR-RTs, a group of TEs, are constituted by *gag pol* genes flanking with long terminal repeat sequences. The *gag* gene encodes the virus-like particle structural proteins where reverse transcription takes place while the *pol* genes encode several enzymes, the protease cleaving the POL polyprotein, the reverse transcriptase copying the retrotransposon RNA into cDNA, and the ribonuclease H domain and an integrase integrating the cDNA into the genome. Under HC conditions compared to LC, Phatr3_J48048 and Phatr3_EG00052 encoding reverse transcriptase were up-regulated, and genes encoding integrase core domain (Phatr3_EG00775, Phatr3_J39341, and Phatr3_Jdraft1453 and Phatr3_J33646) also showed up-regulation. In addition, 17 genes encoding putative endonuclease or exonuclease were up-regulated in HC compared to LC. These results demonstrated that TEs were active in response to elevated *p*CO_2_.

**FIGURE 2:**
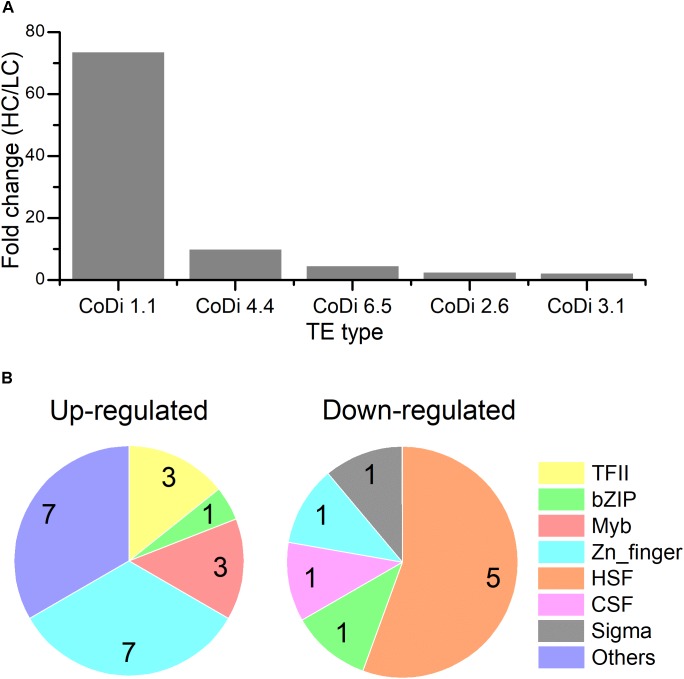
**(A)** The expression levels of different classes of transposon elements (HC/LC) after *P. tricornutum* grown for 15 generations. **(B)** Pie charts of different classes of up-regulated and down-regulated transcription factors (TFs) under the HC condition compared to the LC condition in *P. tricornutum* after grown for 15 generations (up-regulated: HC/LC fold change ≥ 1.5, down-regulated: HC/LC fold change ≤ 0.67, *p*_adj_ < 0.05).

The LTR-RTs are classified into *Ty1/copia* elements and *Ty3/gypsy* elements based on the organization of their *pol* genes and similarities among their encoded reverse transcriptase proteins. Among these active TEs, *Copia* type retrotransposons were the most active transposons in the HC condition compared to LC based on the order of core domain in the *pol* region. Based on the TE sequences provided in (Maumus et al., 2009), CoDi 1.1 (EU432476.1, Phatr3_EG00052), CoDi 4.4 (EU432484.1, Phatr3_EG01511), CoDi 6.5 (EU432496.1, Phatr3_EG00775), CoDi 2.6 (EU363804.1, Phatr3_J50428), and CoDi 3.1 (EU432481.1, Phatr3_J33646) were significantly up-regulated in response to the HC condition, as shown in Figure 2. In *P. tricornutum*, it has been reported that *Blackbeard* and *Surcouf*, belonging to the *Ty1/copia* TEs, were responsive to nitrate starvation and high decadienal condition, respectively (Maumus et al., 2009). In our study, the activation of *Copia* TEs, especially CoDi 1.1 which was the most active TE, in response to the HC condition after growing for 15 generations was consistent with what has been observed in natural environmental samples that *Copia* TEs were more active compared to other TEs (Lescot et al., 2016). This suggests that certain TEs specifically respond to given conditions and further emphasize the importance of *Copia* TEs in response to different environmental stimuli.

The mobile nature of TEs can contribute to alterations in genetic regulatory elements, therefore altering levels of gene expression, triggering genome rearrangements and mutations that can accelerate biological diversification and consequently influence genome evolution (Fedoroff, 2012; Lisch, 2013). It is generally considered that the insertion of TEs has detrimental effects, including gene inactivation and alteration of chromosome structure. On the other hand, the beneficial effects of TEs have also been noted. TEs can enhance their hosts’ fitness, induce advantageous rearrangements or enrich the host’s gene pool in the long term (Schaack et al., 2010; Casacuberta and González, 2013). It has been further recognized that TEs as diversifying agents may contribute to adaptive processes occurring over short time scales. These aspects may explain the abundance and ubiquity of TEs in the natural environment (Lisch, 2013; Belyayev, 2014; Schrader et al., 2014).

In our study, the activation of TEs accompanying the down-regulation of histone genes in the HC condition may increase the potential for genome restructuring, and consequently to reshape gene expression patterns by TE insertions in promoters, enhancers and exons, and result in sequence expansion, gene duplication, novel gene formation and expansion, and re-wiring of genetic regulatory networks. More importantly, the effects on the genomes induced by elevated *p*CO_2_ on the acclimated state could be transmissible to future generations, which not only increase the fitness and phenotypic variation for acclimation in short time but also tremendously influence the adaptation and evolution of the whole population, such as the whole diatom community, in the long term under the elevated *p*CO_2_ conditions (Figure 6). Accumulating data has shown that TEs are not only the most abundant and ubiquitous genes in nature but also are transcriptionally active in marine assemblages and in mutualistic endosymbionts, which further indicate their importance in response to environmental change (Aziz et al., 2010; Kleiner et al., 2013; Lescot et al., 2016).

DNA methylation and histone modifications are two major epigenetic components, which can mediate heritable changes in gene functions beyond DNA sequence changes. In *P. tricornutum*, cytosine DNA methylation is commonly found in TEs and may be involved in controlling TE mobility in the genome (Veluchamy et al., 2013). As a case in point, the activation of the retrotransposon *Blackbeard* was accompanied by its hypomethylation under nitrate starvation (Maumus et al., 2009). The “epi-transposon” hypothesis proposes that changing environments can lead to stress-induced breakdown of epigenetic suppression of TEs (such as DNA methylation), which results in extensive transposition, thus providing new material for rapid adaptive shifts in short term acclimation (Zeh et al., 2009). It was reported that some TEs lose DNA methylation in *P. tricornutum* in response to nitrate starvation (Veluchamy et al., 2015). In our study, besides the down-regulation of histone genes, the burst of TEs was the most significant genetic response in the acclimated HC state. We therefore speculate that the highly expressed TEs may be accompanied by DNA demethylation on TEs in response to elevated *p*CO_2_, although there is currently a lack of genome wide DNA methylation data under the HC and LC conditions. It has been shown that reducing the amount of epigenetic variation available to populations can reduce adaptation in the green alga *Chlamydomonas reinhardtii*, which highlights the importance of epigenetic changes in the adaptation response to environmental changes (Kronholm et al., 2017).

Previous studies have also shown that histone modification may play a role in responses to different environmental stimuli in diatoms. Histone acetylation is usually considered as the positive hallmark for gene expression and H3K9/14 acetylation and H3K4me2 marks were found to be modulated in response to nitrate limitation in *P. tricornutum* (Veluchamy et al., 2015). It was further reported that genes encoding putative proteins involved in epigenetic modifications were up-regulated under phosphorus depletion in *P. tricornutum* (Cruz de Carvalho et al., 2016). In this study, the genes encoding proteins responsible for histone acetylation (Phatr3_J45764, Phatr3_J54343, Phatr3_J45703, Phatr3_EG02442, and Phatr3_J45644) were up-regulated in HC. Further studies on whether the up-regulation of histone acetylases leads to acetylation of specific histones residues resulting in up-regulation of histone acetylation marked genes under elevated *p*CO_2_ conditions in *P. tricornutum* will therefore be of interest.

**FIGURE 3:**
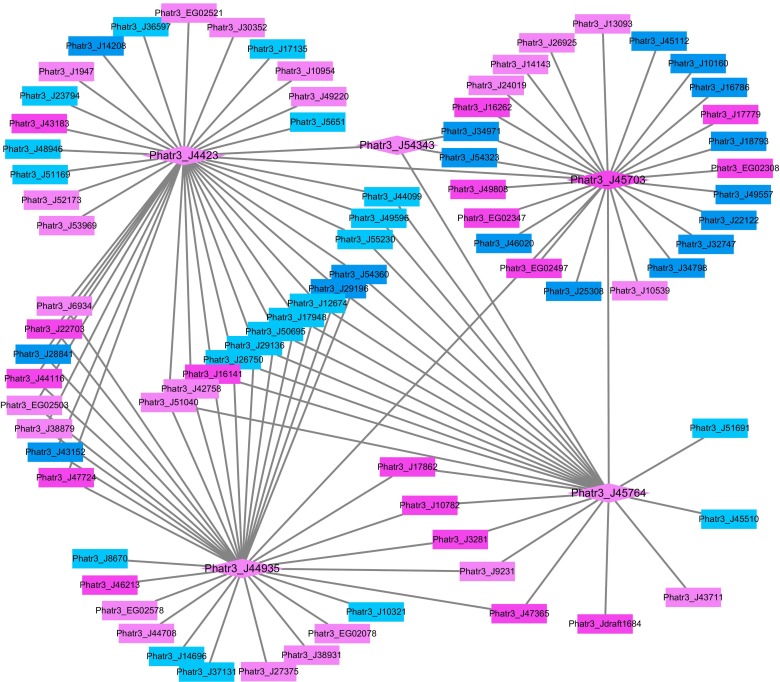
The network of differentially expressed histone modification genes and their correlated differentially expressed mRNAs under the HC relative to the LC conditions after growing for 15 generations. The diamonds illustrate histone modification genes while the rectangles illustrate other mRNAs. The up-regulated genes are filled with pink color and the down-regulated genes are filled with blue color (UGs: dark pink, DGs: dark blue, RUGs:light pink, RDGs:light blue).

In order to further analyze the potential roles of histone modification in response to HC, the network of differentially expressed putative histone modification genes and their associated genes was constructed in Cytoscape (Figure 3). All putative histone modification genes, including putative histone deacetylase Phatr3_J4423, putative histone acetyltransferases Phatr3_J45703, Phatr3_J54343, and Phatr3_J45764 and putative H3K4 methyltransferase Phatr3_J44935 were up-regulated in the constructed network in HC. The putative H3K4 methyltransferase (Phatr3_J44935), histone deacetylase genes (Phatr3_J4423) and histone acetyltransferases (Phatr3_J45703 and Phatr3_J45764) are highly connected with other genes, which implies that H3K4 methylation and histone deacetylation or/and acetylation may be involved in regulating genes in response to elevated *p*CO_2_, such as genes involved in TCA cycle and carbon assimilation (Phatr3_J53935, Phatr3_J22122).

### The Response of Transcription Factors to Elevated *p*CO_2_

The TFs of diatoms are able to change their activity and ultimately control the diatom transcriptomic response to different signals. We investigated the expression profile of 289 genes encoding putative TFs (Rayko et al., 2010; Cruz de Carvalho et al., 2016). Among them, 30 TFs (21 up-regulated TFs with HC/LC fold change ≥ 1.5 and 9 down-regulated TFs with HC/LC fold change ≤ 0.67) were differentially expressed after growing for 15 generations in HC compared to LC (Supplementary Table S7 and Figure 2). Phatr3_J37556 encoding transcription initiation factor TFIIB was the most up-regulated TF while Phatr3_J45112 encoding a heat shock factor was the most down-regulated TF. A total of seven up-regulated TFs belonged to Zn-finger and more than 50% up-regulated TFs were C2H2-type. In addition, three up-regulated TFs belonged to “Myeloblastosis” family, which may mediate signal transduction pathway in response to abiotic drivers (Chen et al., 2006). Meanwhile, 50% of the down-regulated TFs belong to heat shock factors (HSFs) which can bind to the conserved heat shock elements found in the promoters of target genes, including heat shock proteins (HSPs) (Åkerfelt et al., 2010). The down-regulation of HSFs correlated with the decreased expression of HSPs, an important group of molecular chaperones involved in protein assembly and folding, suggesting an acquired acclimated state under elevated pCO_2_.

### Long Non-coding Transcripts Specifically Induced by Elevated *p*CO_2_

**FIGURE 4:**
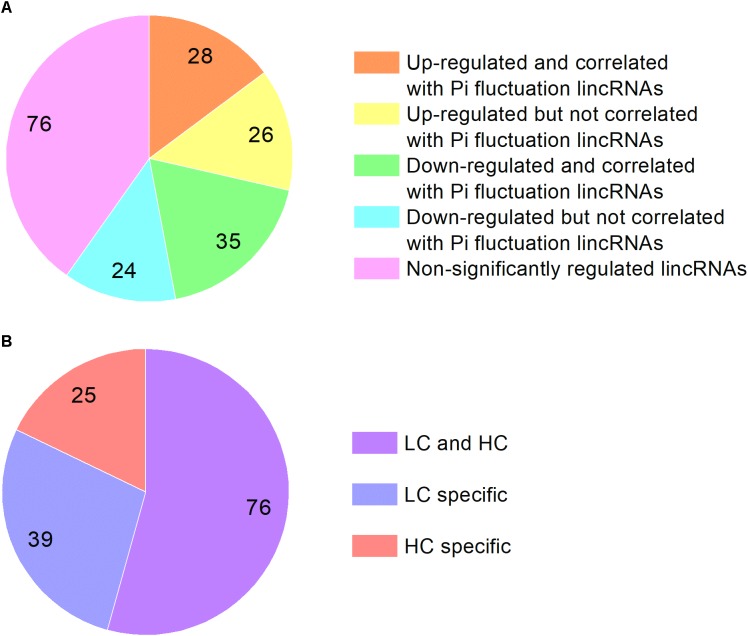
**(A)** The classification of lincRNAs identified in this study based on the correlation with lincRNAs identified in phosphate fluctuation study and expression. **(B)** The classification of lincRNAs identified in phosphate fluctuation study based on the correlation with elevated CO_2_ responsive lincRNAs.

We analyzed the *P. tricornutum* non-coding transcriptome and identified 189 lncRNAs which are all intergenic lncRNAs (lincRNAs). The majority of the lincRNAs identified are between 600 and 700 nt in length and are significantly shorter than mRNAs, which range from 100 nt to more than 3000 nt. The majority of the detected *P. tricornutum* lincRNAs were also intronless, with one exon of similar size to the exons found in mRNAs previously described (Cruz de Carvalho et al., 2016) (Supplementary Figure S2). This is a common feature of lincRNAs and has been suggested to be related to the nuclear localization of these transcripts (Niazi and Valadkhan, 2012). The expression levels of lincRNAs were lower than mRNA in *P. tricornutum* as shown in Supplementary Figure S3 which is also a common feature of this class of transcripts. Among them, 54 lincRNAs were up-regulated in HC while 59 lincRNAs were down-regulated. 28 up-regulated lincRNAs and 35 down-regulated lincRNAs are correlated with lincRNAs identified in the phosphate fluctuation study (Cruz de Carvalho et al., 2016) (Figure 4). The most significantly up-regulated lincRNA was lnc_117 while the most down regulated lincRNA was lnc_145 (Supplementary Table S8).

**FIGURE 5:**
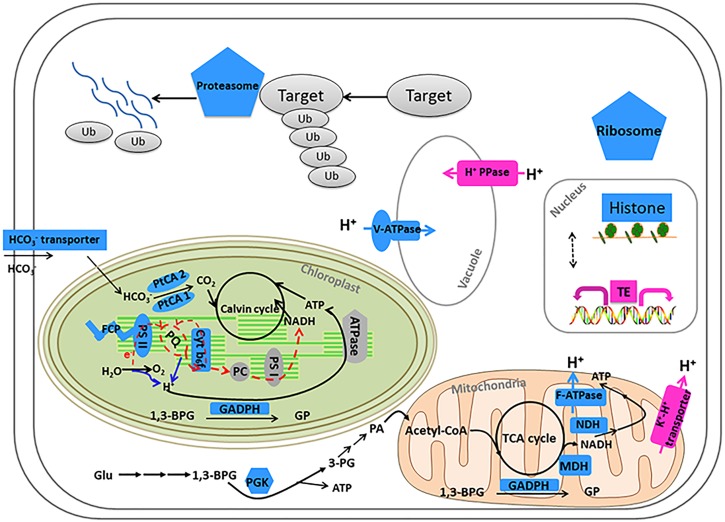
Model of metabolic and signaling pathways in *P. tricornutum* after acclimation to HC for 15 generations. Gene expression changes (HC/LC) are indicated by different colors: pink (up-regulation) and blue (down-regulation). Solid arrows indicate reactions and dashed arrows indicate regulatory relationships. HCO_3_^-^ transporter: Phatr3_J54405; Fucoxanthin chlorophyll a-c binding protein or Fucoxanthin chlorophyll a/c protein (FCP): Phatr3_J24119, Phatr3_J25893, Phatr3_J18049, Phatr3_J27278, Phatr3_J54027; Photosystem II subunit (PS II): Phar3_J55057, Phatr3_J44899; Carbonic anhydrase (PtCA 2): Phar3_J45443; Carbonic anhydrase (PtCA 1): Phar3_J51305; Cyt_b6f_ iron-sulfur subunit: Phatr3_J13358; NADH dehydrogenase (NDH): Phatr3_EG00870, Phatr3_EG01423; Malate dehydrogenase, mitochondrial (MDH): Phar3_J42398; Mitochondrial ATPase: Phar3_J39529; F-ATPase: Phatr3_J31133; 1,3-BPG: 1,3-bisphosphoglycerate; 3-PG: 3-phosphoglycerate; GP: Glyceraldehyde 3-phosphate; Glyceraldehyde 3-phosphate dehydrogenase (GADPH): Phar3_J25308 (mitochondria), Phar3_J22122 (chloroplast), Phar3_J32747; Glu: Glucose; PA: pyroracemic acid; Phosphoglycerate kinase (PGK): Phar3_J48983; Histones: Phatr3_J34971, Phatr3_J54360, Phatr3_J46020, Phatr3_J11841, Phatr3_J26896, Phatr3_J26802, Phatr3_J34798, Phatr3_J50872, Phatr3_J11823, Phatr3_EG02092, Phatr3_EG01358, Phatr3_J50695; Ribosome: Phatr3_J17519, Phatr3_Jdraft477, Phatr3_J28562, Phatr3_J10196, Phatr3_J47804, Phatr3_J36226, Phatr3_J51066; Proteasome: Phatr3_Jdraft611, Phatr3_Jdraft866, Phatr3_EG00973, Phatr3_J30003, Phatr3_J5685, Phatr3_EG02026, Phatr3_J27508, Phatr3_J49897, Phatr3_J51691, Phatr3_J20007, Phatr3_EG02638, Phatr3_J24474; V-ATPase: Phatr3_J31133; Inorganic H^+^ pyrophosphatase (H^+^ PPase): Phatr3_J43207; K^+^-H^+^ transporter: Phatr3_J50171.

We compared the lincRNAs identified in our study with the lincRNAs associated with a phosphate fluctuation response (Cruz de Carvalho et al., 2016). The results showed that 140 lincRNAs associated with phosphate fluctuations are also responsive to CO_2_ concentrations. Among these 140 lincRNAs, 25 lincRNAs are associated HC conditions, 39 lincRNAs are associated with LC conditions, and 76 lincRNAs are associated with both HC and LC conditions as shown in Supplementary Table S9. Most of the lincRNAs identified in this study are also involved in phosphate stress responses suggesting that linRNAs may play central regulatory roles in response to different environmental conditions. LincRNAs have been described previously to be involved in a multitude of regulatory processes at the transcriptional, post-transcriptional and epigenetic levels (Niazi and Valadkhan, 2012; Amaral et al., 2013; Hon et al., 2017). LincRNAs have been namely reported to associate with several regulatory proteins such as TF and chromatin modifying complexes and have central roles in cellular homeostasis and stress response adaptations (Amaral et al., 2013). Although the regulatory roles of lincRNAs in *P. tricornutum* in response to environmental stimuli remain to be determined, their expression patterns under HC and phosphate fluctuations indicate a tight regulation, which strongly suggests function.

## Conclusion

**FIGURE 6:**
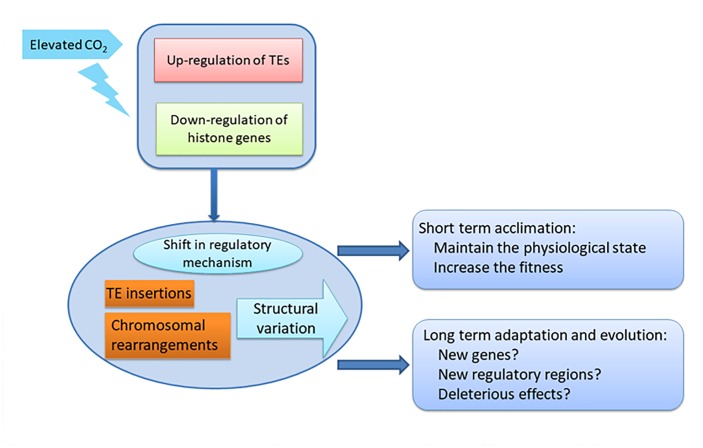
Schematic of the impact of down-regulation of histone genes and up-regulation of TEs on acclimation and adaptation of diatoms under elevated CO_2_.

Our results suggest protectthat chromatin-based processes may be important for the response of *P. tricornutum* to ocean acidification, not only in short term acclimation but also in long term adaptation (Figure 6). The striking activation of TEs combined with down regulation of histone genes indicates the potential epigenetic component in the response to ocean acidification in *P. tricornutum*. Some studies have shown that the dynamic of histone modifications are accompanied with the changes of histone genes expression (Busslinger et al., 1985; Rao et al., 2007; Zager and Johnson, 2009). This indicates that ocean acidification may profoundly influence the adaptive processes of diatoms, important primary producers in the oceanic environment with important impacts in marine ecosystems. It was proposed that when organisms are challenged by new conditions, they can improve their fitness by a multitude of processes involving physiological acclimation, epigenetic changes, structural re-arrangements of the genome, and changes in DNA sequence, described as the adaptation spectrum. Different adaptations are characterized by the time needed for organisms to attain them and by their duration (Yona et al., 2015). Physiological adaptation is what most studies focus on, but while they may confer selective advantages, they are not actively amplified, memorized, or propagated over many generations. On the next level are epigenetic adaptations and DNA copy-number adaptations, which constitute a molecular “memory” of relatively labile genetic changes, although they do not involve changes in the actual nucleotide sequence of the genome. The observed transcriptional activation of TEs and the down-regulation of histone genes may induce further changes that influence the long term adaptation of *P. tricornutum,* such as after growing 1000 or longer generations under elevated *p*CO_2_ (Figure 6). In contrast with physiological adjustments, this level of adaptation could be transmitted to subsequent generations and thus have more profound effects on the whole community. New genes and new regulatory elements may appear in *P. tricornutum* due to potential TE insertions and chromosomal arrangement, which may lead to new metabolic state in response to elevated *p*CO_2_ as reported in Li et al. (2017). On the other hand, TE insertions and chromosomal arrangement may also lead to deleterious effects in *P. tricornutum*. The importance of epigenetic regulation in response to environmental changes in *P. tricornutum* may be extrapolated to the whole diatom community.

In order to better understand the effects of ocean acidification, in addition to the ecological responses, the evolutionary responses and the underlying molecular mechanisms should be taken into account. It will be very interesting and worthwhile to investigate how epigenetic regulation and variation contribute to the acclimation in short term and adaptation in long term in response to elevated *p*CO_2_ using epigenetic approaches, such as genome wide methylome analysis and histone modification analysis (such as histone acetylation) by ChIP-seq. Furthermore, the expression profiles of several lincRNAs also suggests their involvement in the acclimation to elevated *p*CO_2_ responses which is worth to be further investigated in the future.

## Data Availability Statement

The sequencing data using in this study have been deposited in the NCBI (PRJNA484278).

## Author Contributions

XL, RH, and KG planned and designed the research. RH performed the experiments. RH, JD, and XL analyzed the data. XL, RH, MCC, CB, and LT wrote the manuscript.

## Conflict of Interest Statement

The authors declare that the research was conducted in the absence of any commercial or financial relationships that could be construed as a potential conflict of interest.

## Footnotes

1 http://protists.ensembl.org/Phaeodactylum_tricornutum/Info/Index

2 http://www.geneontology.org

3 http://www.kegg.jp/

4 http://string-db.org/
