# Corrigendum: A Potential Role for Epigenetic Processes in the Acclimation Response to Elevated *p*CO_2_ in the Model Diatom *Phaeodactylum tricornutum*

**DOI:** 10.3389/fmicb.2019.00158

**Published:** 2019-02-08

**Authors:** Ruiping Huang, Jiancheng Ding, Kunshan Gao, Maria Helena Cruz de Carvalho, Leila Tirichine, Chris Bowler, Xin Lin

**Affiliations:** ^1^State Key Laboratory of Marine Environmental Science, College of Ocean and Earth Sciences, Xiamen University, Xiamen, China; ^2^School of Pharmaceutical Sciences, Xiamen University, Xiamen, China; ^3^Ecology and Evolutionary Biology Section, Institut de Biologie de l'École Normale Supérieure (IBENS), Département de Biologie, Ecole Normale Supérieure, CNRS UMR8197, Inserm U1024, PSL Research University, Paris, France; ^4^Faculté des Sciences et Technologie, Université Paris Est-Créteil, Créteil, France; ^5^Faculté des Sciences et Techniques, Université de Nantes, CNRS UMR6286, UFIP, Nantes, France

**Keywords:** ocean acidification, climate change, diatom, transposable element, histone, long non-coding RNA

In the published article, there was an error regarding the affiliation for Maria Helena Cruz de Carvalho. As well as having affiliation 3, they should also have “Faculté des Sciences et Technologie, Université Paris Est-Créteil, Créteil, France.”

The authors apologize for this error and state that this does not change the scientific conclusions of the article in any way. The original article has been updated.

## Conflict of Interest Statement

The authors declare that the research was conducted in the absence of any commercial or financial relationships that could be construed as a potential conflict of interest.

