# Supplementary material for: A Potential Role for Epigenetic Processes in the Acclimation Response to Elevated pCO2 in the Model Diatom Phaeodactylum tricornutum
Source: Front Microbiol. 2019 Jan 14;9:3342. doi: 10.3389/fmicb.2018.03342 (PMC6340190; doi:10.3389/fmicb.2018.03342)
Supplement: Supplementary file 1 [file Table_1.DOCX]

|  | *p*CO2(μatm) | pH_NBS_ | DIC(μmol/kg) | HCO_3_^-^( μmol/kg) | CO_3_^2-^( μmol/kg) | TA |
| --- | --- | --- | --- | --- | --- | --- |
| LC_before_ | 436.76±9.12 | 8.14±0.01 | 2037.19±6.82 | 1852.30±8.52 | 170.78±2.00 | 2274.35±3.45 |
| LC_after_ | 457.40±7.74 | 8.11±0.01 | 1989.12±6.61 | 1817.37±7.41 | 156.98±1.59 | 2207.57±5.27 |
| HC_before_ | 1042.66±17.97 | 7.80±0.01 | 2140.14±1.21 | 2021.28±1.94 | 85.19±1.31 | 2232.36±1.28 |
| HC_after_ | 1014.37±23.06 | 7.81±0.01 | 2131.70±12.87 | 2012.16±12.98 | 86.77±0.85 | 2227.27±10.66 |
