# Supplementary material for: A Potential Role for Epigenetic Processes in the Acclimation Response to Elevated pCO2 in the Model Diatom Phaeodactylum tricornutum
Source: Front Microbiol. 2019 Jan 14;9:3342. doi: 10.3389/fmicb.2018.03342 (PMC6340190; doi:10.3389/fmicb.2018.03342)
Supplement: Supplementary file 10 [file Table_10.DOCX]

**Supplementary table 1** The average values of parameters of carbonate system before and after the dilution.

|  | *p*CO2(μatm) | pH_NBS_ | DIC(μmol/kg) | HCO_3_^-^( μmol/kg) | CO_3_^2-^( μmol/kg) | TA |
| --- | --- | --- | --- | --- | --- | --- |
| LC_before_ | 436.76±9.12 | 8.14±0.01 | 2037.19±6.82 | 1852.30±8.52 | 170.78±2.00 | 2274.35±3.45 |
| LC_after_ | 457.40±7.74 | 8.11±0.01 | 1989.12±6.61 | 1817.37±7.41 | 156.98±1.59 | 2207.57±5.27 |
| HC_before_ | 1042.66±17.97 | 7.80±0.01 | 2140.14±1.21 | 2021.28±1.94 | 85.19±1.31 | 2232.36±1.28 |
| HC_after_ | 1014.37±23.06 | 7.81±0.01 | 2131.70±12.87 | 2012.16±12.98 | 86.77±0.85 | 2227.27±10.66 |

**Supplementary table 2** The genes involved in chromatin, protein dna, chromosomal part, chromosome, histone modification, glycolysis TCA cycle, photosynthesis(light part), carbon assimilation and nitrogen uptake and assimilation are classified based on the annotation and GO analysis.

**Supplementary table 3** The primers used for q-PCR validation

**Supplementary table 4** The mapping rate reads from ssRNA-seq data generated at 15 generation.

**Supplementary table 5** The expression level of each gene under the HC and the LC conditions after growing for 15 generations.

**Supplementary table 6** The results of q PCR validation.

**Supplementary table 7** The differentially expressed TFs under the HC and LC conditions after growing for 15 generations.

**Supplementary table 8** The differentially expressed lincRNAs under the HC and LC conditions.

**Supplementary table 9** Sequence comparison of lincRNAs in this study with linRNAs under phosphate limitation conditions.
