# Supplementary material for: A Potential Role for Epigenetic Processes in the Acclimation Response to Elevated pCO2 in the Model Diatom Phaeodactylum tricornutum
Source: Front Microbiol. 2019 Jan 14;9:3342. doi: 10.3389/fmicb.2018.03342 (PMC6340190; doi:10.3389/fmicb.2018.03342)

Supplementary Figure 1 The GO enrichment analysis of DEGs in the domains of biological processes, cellular components and molecular functions at 15 generation.

Supplementary Figure 2 Genomic structure of *P. tricornutum* long noncoding RNAs (lncRNAs). (a) Exon numbers of unspliced lincRNAs and mRNA. (b) Length distribution in nucleotides (nt) of unspliced lncRNAs and mRNAs;

Supplementary Figure 3 The comparison of expression levels of mRNA and LncRNA in HC and LC samples.

Supplementary Figure 1

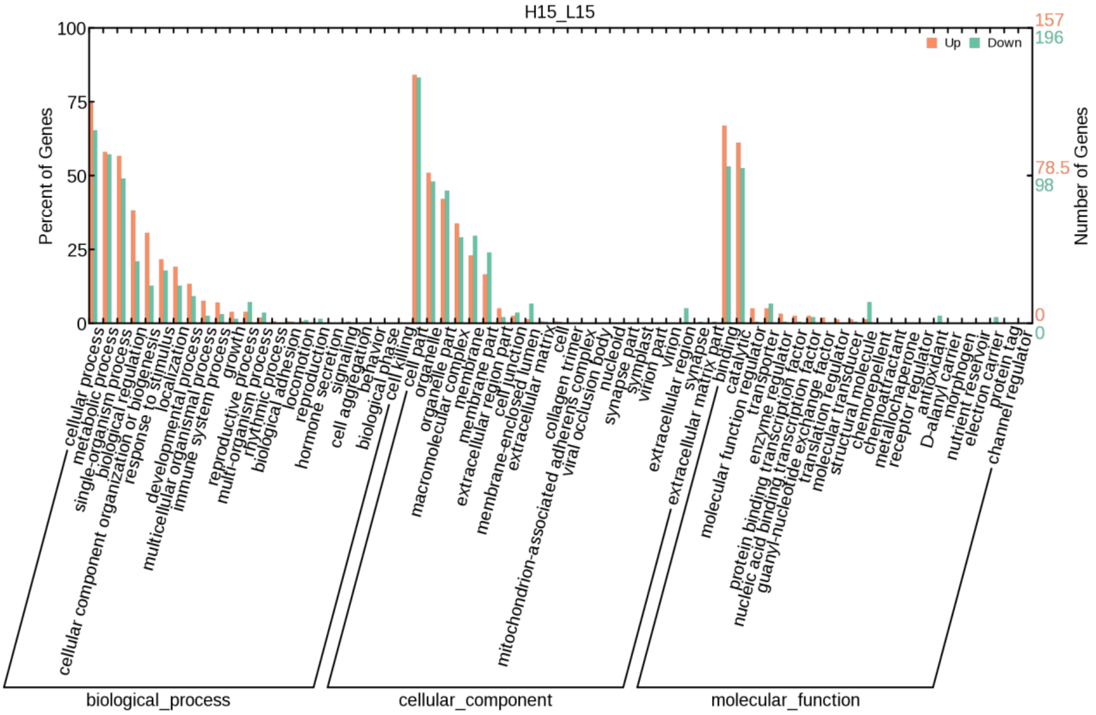

Supplementary Figure 2

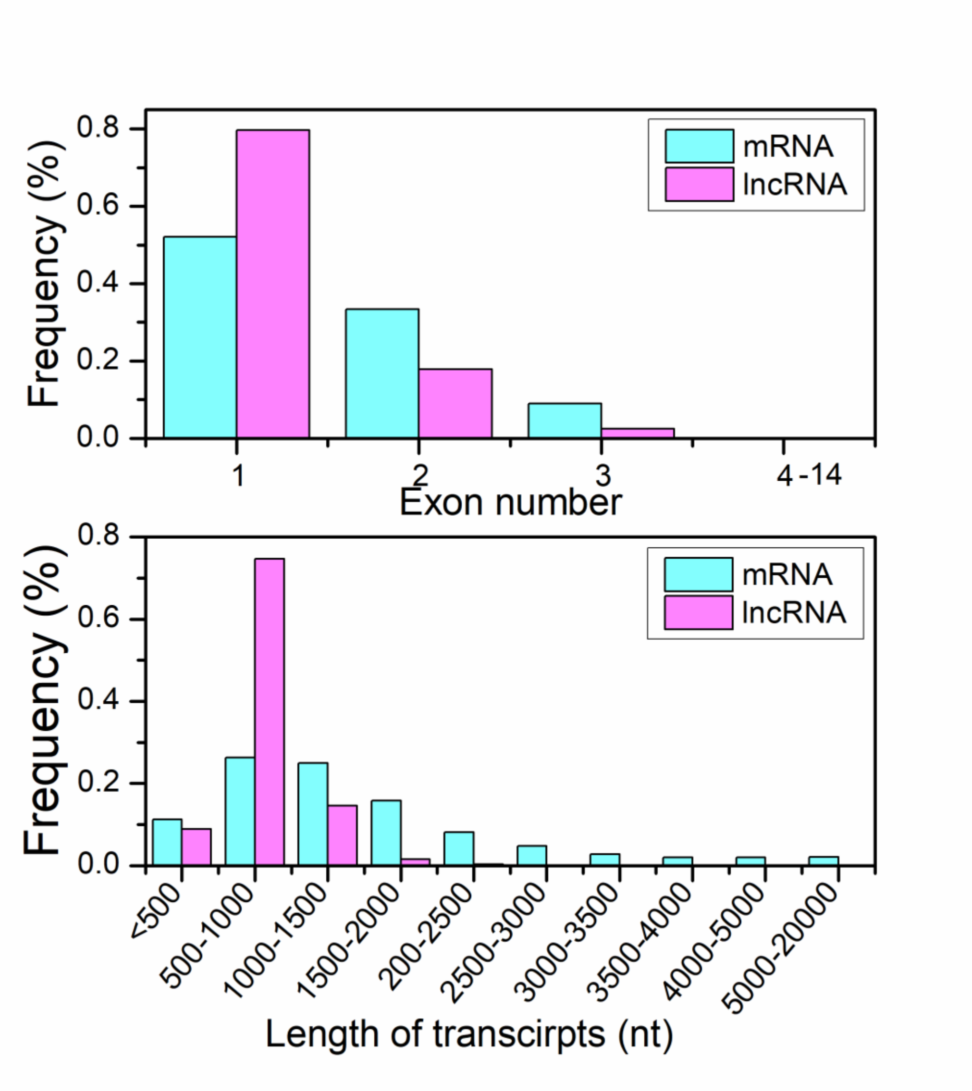

Supplementary Figure 3

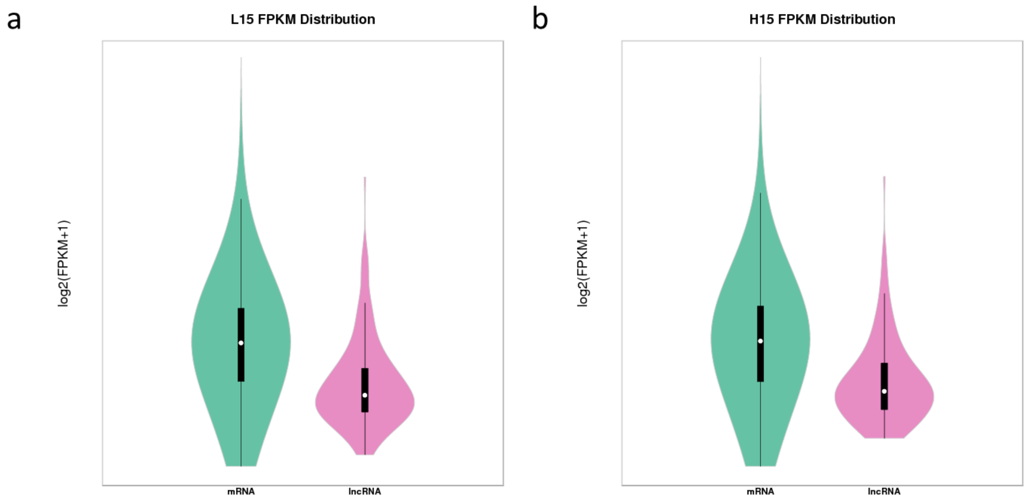

Supplement: Supplementary file 11 [file Data_Sheet_1.pdf]
